# Herpes Simplex Virus 1 and Cytomegalovirus infections and structural brain imaging markers in the UK Biobank

**DOI:** 10.1002/alz70860_106765

**Published:** 2025-12-23

**Authors:** Marie‐Anne Pietrowski, Fabrice Crivello, Sonia Burrel, Jean‐François Dartigues, Catherine Helmer, Morgane Linard

**Affiliations:** ^1^ University of Bordeaux, INSERM, BPH, U1219, Bordeaux, France; ^2^ University of Bordeaux, CNRS, CEA, IMN, GIN, Bordeaux, France; ^3^ Bordeaux University Hospital, Bordeaux, France; ^4^ University of Bordeaux, MFP, CNRS, UMR 5234, Bordeaux, France; ^5^ University of Bordeaux, INSERM, Bordeaux Population Health Research Center, UMR U1219i, City of Bordeaux, Bordeaux, France

## Abstract

**Background:**

While previous studies have suggested the involvement of herpes simplex virus 1 (HSV‐1) in the pathophysiology of Alzheimer's disease (AD), neuroimaging studies in this area are scarce. We aimed to determine the association of HSV‐1 infection with neuroimaging markers of AD and to assess the impact of susceptibility factors that could modulate the deleterious effects of HSV‐1.

**Method:**

Within a subsample of the UK Biobank with both serological and MRI data, we analyzed the associations between HSV‐1 seropositivity and grey matter (GM) volumes in four brain areas affected early in AD (hippocampus, amygdala, parahippocampal and entorhinal cortex), using linear regressions adjusted on potential cofounders (age, sex, APOE4 allele, education, income, smoking and alcohol intake, diabetes, hypertension, body mass index and total intracranial volume). To assess the impact of susceptibility factors, interactions of HSV‐1 with cytomegalovirus (CMV), APOE4 and age were tested and stratifications on these factors were performed. CMV infection and co‐infection with HSV‐1 were further investigated to identify the separate and cumulative effects of each virus.

**Result:**

Among 901 participants (mean age 64.1 (SD = 7.7) at MRI; 55.6% of women), when considering only HSV‐1 we found no significant difference in GM volume in the four brain regions studied between HSV‐1‐infected and uninfected participants. Nevertheless, considering interaction with CMV, HSV‐1 infection was associated with a lower parahippocampal volume in CMV non‐infected participants only (β = ‐105 mm^3^, *p* = 0.03). Further investigation of interactions with CMV and other susceptibility factors showed that, among APOE4 carriers ≥ 65y, i.e. the most at‐risk people for AD: i) those infected only by CMV had lower amygdala volume (β = ‐197 mm^3^, *p* = 0.04); and ii) those infected by CMV (alone or in combination with HSV‐1) tended to have smaller GM volumes in three other brain regions studied (*p* < 0.20).

**Conclusion:**

Our study highlighted the complexity of the interactions involved when assessing associations between viral infections and radiological biomarkers of AD and the importance of considering susceptibility factors such as viral co‐infection, age and APOE4 genotype to decipher the role of infections in AD.

